# Identifying Predictive Gene Expression and Signature Related to Temozolomide Sensitivity of Glioblastomas

**DOI:** 10.3389/fonc.2020.00669

**Published:** 2020-05-22

**Authors:** Hong-Qing Cai, Ang-Si Liu, Min-Jie Zhang, Hou-Jie Liu, Xiao-Li Meng, Hai-Peng Qian, Jing-Hai Wan

**Affiliations:** ^1^Department of Neurosurgery, National Cancer Center/National Clinical Research Center for Cancer/Cancer Hospital, Chinese Academy of Medical Sciences and Peking Union Medical College, Beijing, China; ^2^Department of Neurosurgery, The Second Affiliated Hospital, Anhui Medical University, Hefei, China

**Keywords:** temozolomide, glioblastoma, gene expression signature, prognosis, telomerase

## Abstract

Temozolomide (TMZ) is considered a standard chemotherapeutic agent for glioblastoma (GBM). Characterizing the biological molecules and signaling pathways involved in TMZ sensitivity would be helpful for selecting therapeutic schemes and evaluating prognosis for GBM. Thus, in the present study, we selected 34 glioma cell lines paired with specific IC_50_ values of TMZ obtained from CancerRxGene and RNA-seq data downloaded from the Cancer Cell Line Encyclopedia to identify genes related to TMZ sensitivity. The results showed that 1,373 genes were related to the response of GBM cells to TMZ. Biological function analysis indicated that epithelial–mesenchymal transition, Wnt signaling, and immune response were the most significantly activated functions in TMZ-resistant cell lines. Additionally, negative regulation of telomere maintenance via telomerase was enriched in TMZ-sensitive glioma cell lines. We also preliminarily observed a synergistic effect of combination treatment comprising TMZ and a telomerase inhibitor *in vitro*. We identified six genes (*MROH8, BET1, PTPRN2, STC1, NKX3-1*, and *ARMC10*) using the random survival forests variable hunting algorithm based on the minimum error rate of the gene combination and constructed a gene expression signature. The signature was strongly related to GBM clinical characteristics and exhibited good prognosis accuracy for both The Cancer Genome Atlas (TCGA) and Chinese Glioma Genome Atlas (CGGA) datasets. Patients in the high score group had a shorter survival time than those in the low score group (11.2 vs. 22.2 months, hazard ratio = 7.31, *p* = 4.59e−11) of the TCGA dataset. The CGGA dataset was selected as a validation group with 40 patients in the high score set and 43 patients in the low score set (12.5 vs. 28.8 months, hazard ratio = 3.42, *p* = 8.61e−5). Moreover, the signature showed a better prognostic value than *MGMT* promoter methylation in both datasets. We also developed a nomogram for clinical use that integrated the TMZ response signature and four other risk factors to individually predict patient survival after TMZ chemotherapy. Overall, our study provides promising therapeutic targets and potential guidance for adjuvant therapy of GBM.

## Introduction

Temozolomide (TMZ) is a DNA-alkylating agent that damages various cellular biological processes by inducing the production of *N*^7^-methylguanine, *N*^3^-methyladenine, and *O*^6^-methylguanine (1). Many clinical studies have verified its strong anticancer effects in glioblastoma (GBM) (2–7). Thus, TMZ has been widely accepted and is highly recommended by guidelines of the National Comprehensive Cancer Network as the standard chemotherapeutic agent for the clinical management of GBM. However, the overall survival and progression-free survival differ significantly among patients subjected to the same TMZ therapy. This is because of the different sensitivities to TMZ in some GBM tissues, which are related to patient molecular backgrounds. Therefore, characterizing the biological molecules or signaling pathways involved in TMZ sensitivity would be helpful for selecting therapeutic schemes and evaluating prognosis for GBM.

Numerous studies have evaluated molecular and signal pathway differences between TMZ-sensitive and TMZ-resistant GBMs. Promoter unmethylation and high expression levels of *O*^6^-methylguanine DNA methyltransferase (MGMT) have been recognized as indicators of the TMZ-resistant phenotype, allowing for more accurate molecular subtyping of tumors (8, 9). Other resistance molecular profiles conferring TMZ resistance to GBMs have also been reported, including overexpression of c-Met, P-glycoprotein, CD133, epidermal growth factor receptor, galectin-1, and Cdc20 proteins (10–13). Several studies indicated that the mechanisms maintaining epithelial–mesenchymal transition (EMT) and stem cell phenotype are also involved in TMZ resistance (14, 15). However, few studies have explored TMZ response-related molecules at the gene transcription level, and no studies have investigated the clinical significance of gene expression signatures related to TMZ sensitivity in glioma.

This study was, therefore, conducted to evaluate genes differentially expressed at the transcriptomic level between TMZ-sensitive and TMZ-resistant GBM cells. GBMs with RNA-seq data and adjuvant TMZ therapy from The Cancer Genome Atlas (TCGA) dataset were selected as a training group to identify genes with significant prognostic value and to find a TMZ response-related gene expression signature, which was validated in the Chinese Glioma Genome Atlas (CGGA) dataset.

## Methods and Materials

### Collection of Publicly Available Data

TMZ sensitivity data from human glioma cell lines were obtained from the CancerRxGene database (16) and for cell lines for which corresponding RNA-seq data were available from the Cancer Cell Line Encyclopedia (17). Thus, a total of 34 cell lines with matched IC_50_ values in the TMZ and RNA-seq profiles were assessed in this study.

Transcript level data and the corresponding clinical parameters of 103 GBMs treated with TMZ were downloaded from TCGA as a training cohort. Data for another 83 GBMs treated with TMZ were obtained from CGGA as an external independent test cohort.

### Cell Culture and Cell Viability Assay

The human glioma cell lines U87MG and U118MG were purchased from the Cobioer Biotechnology Company (Nanjing, China). All cells were cultured in high-glucose Dulbecco's modified Eagle's medium containing 10% fetal bovine serum, 100 mg/ml of streptomycin, and 100 U/ml of penicillin and maintained in a humidified atmosphere containing 5% CO_2_ at 37°C.

Glioma cells from each group were seeded into five wells of a multi-well plate. After overnight serum starvation, the cells were treated with different concentrations of TMZ, BIBR 1532, and their combinations at the indicated dilutions for 72 h. Cell viability was assessed using the Cell Counting kit-8 (CCK-8; Dojindo, Kumamoto, Japan) according to the manufacturer's protocol. The absorbance at 450 nm was measured using a spectrophotometer (BioTek Instruments, Winooski, VT, USA). The inhibition rate was calculated as 1 (absorbance of treatment group/absorbance of control group) × 100%.

### Bioinformatics Analysis

Significantly related genes and their IC_50_ values in glioma cell lines were retrieved by Spearman correlation analysis. Gene Ontology (GO) analysis of these genes was performed using the “ClusterProfiler” package using the R software. In addition, the GESA software was used to reveal the biological processes determined by gene expression patterns of the two groups. Heatmaps and bubble plots were drawn using the “pheatmap” and “ggplot2” packages in the R software.

### Identification of a TMZ Response-Related Signature

The univariate Cox regression analysis was performed to screen out prognosis-related genes with using gene expression and clinical data of GBM patients from the TCGA dataset. Then, random survival forests variable hunting (RSFVH) algorithm further filtered gene combinations. The score of each model was calculated as following: score = β_1_X_1_ + β_2_X_2_ + β_3_X_3_ + …+ β_N_X_N_. N is the number of selected genes of each model, and β is the coefficient of genes in the univariate Cox regression analysis.

### Statistical Analysis

Statistical analysis and the generation of figures were mainly carried out in the statistical programming environment R. The best cut-off points for gene expression and TMZ response-related scores were determined using the “survminer” package with the minimum percentage of the two groups being 30%. The “survival” package was, then, employed to perform log-rank tests and draw survival curves. The “timeROC” package was applied to compare the predictive power of expression signatures and *MGMT* promoter methylation patterns. Student's *t*-test was used to compare differences between groups. Nomograms were constructed using the “rms” package, and calibration plots were generated to evaluate the performance of the nomogram. A value of *P* < 0.05 was considered to indicate statistical significance. All statistical tests were two-tailed.

## Results

### Genes Related to TMZ Response in Glioma Cell Lines

**Figure 1:**
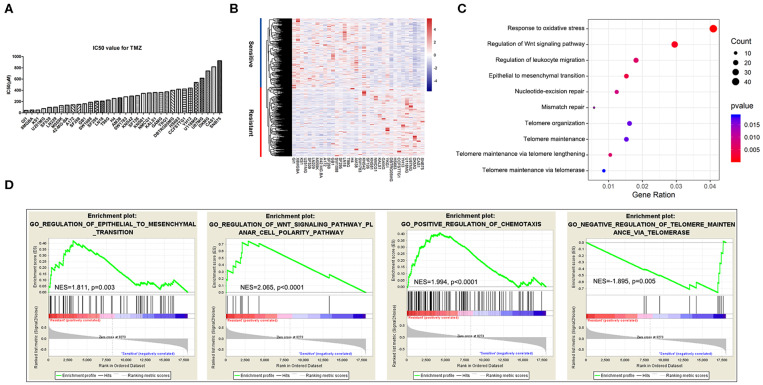
Genes and biological processes related to temozolomide (TMZ) response in glioblastoma (GBM). **(A)** IC_50_ value distribution of 34 glioma cell lines. **(B)** Heatmap of the expression of 1,373 TMZ sensitivity-related genes. **(C)** Enrichment of biological functions related to TMZ sensitivity using GO analysis. **(D)** Biological processes of TMZ response analyzed using the GSEA software.

To comprehensively identify genes related to GBM sensitivity to TMZ chemotherapy, we chose 34 glioma cell lines paired with specific IC_50_ values of TMZ obtained from CancerRxGene and RNA-seq data downloaded from the Cancer Cell Line Encyclopedia. The IC_50_ values ranged from 43.4 to 926 μM, with a median IC_50_ value of 276 μM. The detailed distribution of TMZ sensitivity for the 34 glioma cell lines is shown in Figure 1A. We subsequently performed Spearman correlation analysis between TMZ sensitivity and RPKM (reads per kilobase million) values for gene expression. Genes with a maximum expression level lower than 1 among the 34 cell lines were not considered for further analysis. A total of 1,373 TMZ response-related genes, among which 659 genes were positively correlated with TMZ sensitivity and 714 genes were positively correlated with TMZ resistance, were identified (|R| > 0.3 and *P* < 0.05) across the 34 cell lines. An overview of the expression patterns of the 1,373 genes in the 34 glioma cell lines is presented in Figure 1B. The detailed results for all these genes are shown in Supplementary Table S1.

### TMZ Response-Related Biological Mechanism

To identify the biological processes involved in the response to TMZ, we analyzed the enriched functions of the 1,373 TMZ sensitivity-related genes identified previously by performing GO analysis. The results showed that some genes were strongly related to the basic processes of nucleic acid replication, translation, and transcription. However, TMZ sensitivity-related genes were mainly involved in EMT signaling, cellular response to oxidative stress, telomere maintenance mechanism, DNA damage repair mechanism, immune response, and Wnt signaling (Figure 1C).

We further examined the functions of these genes using the Gene Set Enrichment Analysis software. Sensitivity and resistance of cell lines were defined according to their median IC_50_ values. Again, genes related to EMT (normalized enrichment score; NES = 1.811, *P* = 0.003), Wnt signaling (NES = 2.065, *P* < 0.0001), and positive regulation of chemotaxis and immune cell migration (NES = 1.994, *P* < 0.0001) were most significantly enriched in the TMZ-resistant cell lines. We also identified active enrichment of the negative regulation of telomere maintenance via telomerase (NES = −1.895, *P* = 0.005) in TMZ-sensitive glioma cell lines (Figure 1D).

### Synergistic Effect of TMZ Combined With Telomerase Inhibitor

**Figure 2:**
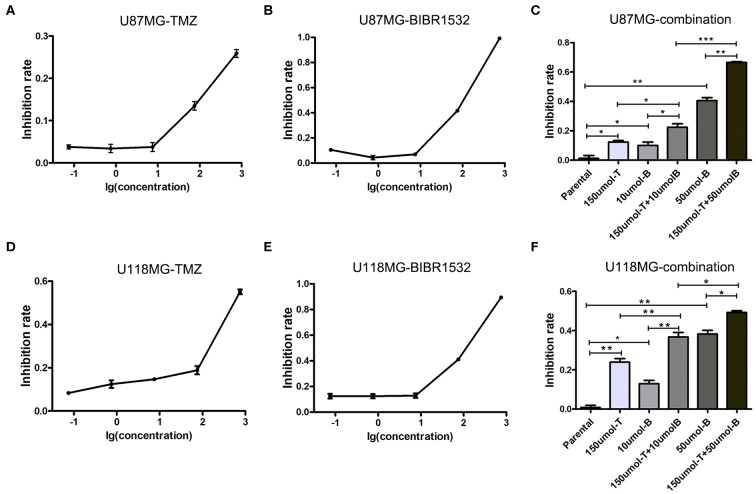
Inhibition of U87MG and U118MG cell lines using TMZ and BIBR1532. **(A–C)** Single treatment and combination treatment with TMZ and BIBR1532 of U87MG cell line. **(D–F)** Single treatment and combination treatment with TMZ and BIBR1532 of U118MG cell line. Data are presented as the mean ± SD. *P < 0.05; **P < 0.01; ***P < 0.001.

Because previous studies demonstrated that the upregulation of telomerase expression and activity following telomerase reverse transcriptase (TERT), promoter mutations comprises the main mechanism through which telomere length is restored (18), we used the telomerase inhibitor BIBR1532 to disrupt telomere maintenance and tested the individual and combinatorial effects of BIBR1532 and TMZ on two glioma cell lines, U87MG and U118MG, with TERT promoter mutations. TMZ and BIBR1532 inhibited cell proliferation in a dose-dependent manner, and U118MG cells were more sensitive than U87MG cells to treatment with the same concentration of TMZ (Figures 2A,B,D,E). Moreover, when the cells were treated with both 10 μM BIBR1532 and 150 μM TMZ, the inhibitory activity of TMZ was significantly stronger than that of each agent alone in the two cell lines (*P* < 0.05 and *P* < 0.01, respectively). Next, we used a higher concentration of BIBR1532 in combination with 10 μM TMZ for treatment. The mean inhibition rates increased to 66.53 and 49.21% in U87MG and U118MG cells, respectively, which were higher than the values for single treatments and lower than that for the combination treatment (Figures 2C,F).

### Foundation of the TMZ Response-Related Signature

To further analyze the prognostic value of the 1,373 TMZ-sensitivity genes identified by us, we performed survival analysis using clinical information and gene expression data for 103 GBM patients treated with TMZ from the TCGA dataset. A total of 116 protein-coding genes and 659 TMZ sensitivity-related genes were found to be associated with favorable survival, whereas 191 protein-coding genes and 714 TMZ resistance-related genes were associated with poor prognosis (*P* < 0.05). We, then, performed dimension reduction using the random survival forests variable hunting algorithm, and screened out six genes (*MROH8, BET1, PTPRN2, STC1, NKX3-1*, and *ARMC10*) from the protein-coding genes related to TMZ response using the minimum error rate of each gene combination (Supplementary Figure S1). These genes were defined as high risk factors with hazard ratios (HRs) > 1. The survival analysis results for these genes are presented in Supplementary Table S2.

We further established the TMZ response-related signature based on the expression status of the abovementioned six high-risk genes based on specific cut-off values and their coefficients as follows: score = 1.36 × status of ARMC10 + 1.19 × status of BET1 + 0.86 × status of MROH8 + 1.47 × status of NKX3.1 + 0.98 × status of PTPRN2 + 1.50 × status of STC1.

### Prognostic Performance of the TMZ Response-Related Signature

**Figure 3:**
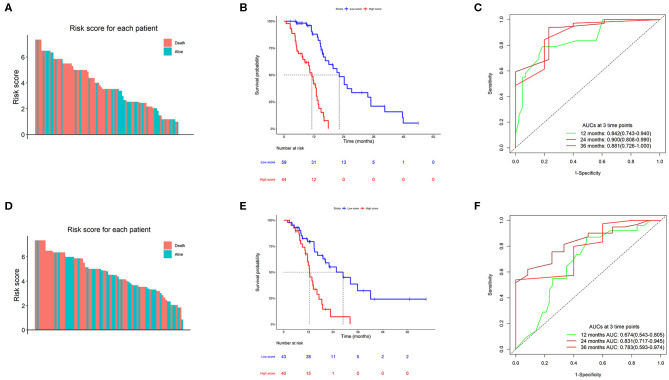
Clinical performance of the TMZ response-related signature. **(A,D)** Risk score of the TMZ response-related signature for all patients in The Cancer Genome Atlas (TCGA) and Chinese Glioma Genome Atlas (CGGA) datasets. **(B,E)** Kaplan–Meier survival charts of high and low scores in the two datasets. **(C,F)** Time-dependent ROC curves at 12, 24, and 36 months in the two datasets.

A total of 103 TMZ-treated GBM patients from the TCGA dataset were divided into two groups, high score (*n* = 44) and low score (*n* = 59) groups, based on the optimum separation cut-off value with minimum p value. The survival analysis of the two groups compared using the log-rank test showed that patients in the high score group had a shorter survival time than those in the low-risk group (11.2 vs. 22.2 months, HR = 7.31, *P* = 4.59e−11). Time-dependent receiver operating characteristic analysis showed that the prognostic accuracies (area under the curve) of the formula were 0.842 (0.743–0.940), 0.900 (0.808–0.990), and 0.881 (0.726–1.000) at 12, 24, and 36 months, respectively (Figures 3A–C). Moreover, 83 TMZ-treated GBM from the CGGA dataset were selected as a validation group for validation of findings obtained using the TCGA dataset, with 40 patients in the high score set and 43 patients in the low score set. Survival analysis results were similar to those for the TCGA dataset, with a median survival time of 12.5 months in the high score group and 28.8 months in the low score group (HR = 3.42, *P* = 8.61e−5). The area under the curve values were 0.674 (95% confidence interval; CI: 0.543–0.805), 0.831 (95% CI: 0.717–0.945), and 0.783 (95% CI: 0.593–0.974) at 12, 24, and 36 months, respectively (Figures 3D–F).

### Independence of the Prognostic Value of TMZ Response-Related Signature

We employed univariate Cox regression analysis to evaluate the survival effect of factors, including gender, age, radiation therapy, *IDH1* mutation, and *MGMT* promoter methylation, which may have contributed to the survival times of 186 patients with GBM. Radiation therapy (HR = 0.279, 95% CI 0.178–0.440, *P* < 0.001) and *MGMT* promoter methylation (HR = 0.611, 95% CI 0.401–0.931, *P* = 0.022) were positively associated with overall survival time. In contrast, age (HR = 1.427, 95% CI 0.973–2.095, *P* = 0.069) and *IDH1* mutation (HR = 0.715, 95% CI 0.412–1.241, *P* = 0.233) were not significantly associated with prognosis. Next, multivariate Cox regression analysis was used to identify independent prognostic indicators for GBM treated with TMZ. The TMZ response-related signature, as well as radiation therapy and *MGMT* promoter methylation, were independently associated with the survival of patients with GBM treated with TMZ (Table 1).

**Table 1:** Univariate and multivariate analyses of variables related to survival.

**Variables**	**Univariate Cox regression**		**Multivariate Cox regression**	
	**HR**	***P* value**	**HR**	***P* value**
Age	1.427	0.069	−	−
Radiation	0.279	<0.001	0.274	<0.001
*MGMT* promoter methylation	0.611	0.022	0.602	0.019
*IDH1* mutation	0.715	0.233	−	−
TMZ response-related signature	1.97	0.001	2.458	<0.001

### Relationship Between TMZ Response-Related Signature and Clinicopathological Features

**Figure 4:**
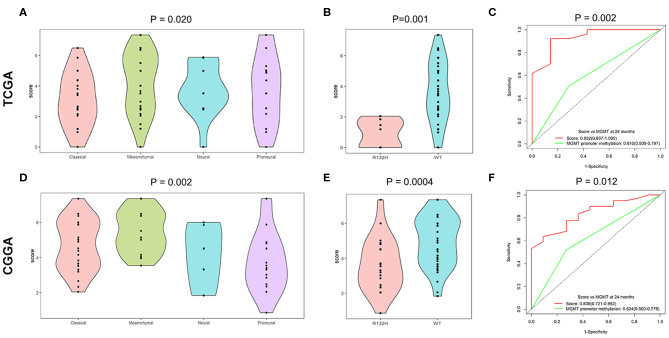
Relationship between score of the TMZ response-related signature and clinicopathological features. **(A,D)** Score of the TMZ response-related signature in classical, neural, and proneural subtypes of the TCGA and CGGA datasets. **(B,E)** The TMZ response model was negatively related to *IDH1* mutation in the two datasets. **(C,F)** Time-dependent ROC curves at 24 months of signature and *MGMT* promoter methylation in both datasets.

To identify the predominant clinicopathological parameters in the TMZ response model, we examined the score differences of the TMZ response model for different clinicopathological types, such as gender, age, molecular subtypes, and *IDH1* mutation. We found that elderly patients (≥ 55 years old) tended to have a high score in both TCGA and the CGGA datasets (*P* = 0.054 and *P* = 0.055, respectively). In contrast, no significant difference between female and male patients in the TCGA and CGGA datasets was observed. In addition, when comparing the classical, neural, and proneural subtypes, the value of the TMZ response signature was significantly higher in the mesenchymal subtype in both TCGA and CGGA cohorts (Figures 4A,D). Moreover, the score for the TMZ response model was negatively associated with *IDH1* mutation. Specifically, the TMZ response signature was highly enriched in *IDH1* wild-type GBMs in both TCGA and CGGA datasets (*P* = 0.001 and *P* = 0.0004, respectively) (Figures 4B,E).

Because *MGMT* promoter methylation is considered as classical biomarker predicting a favorable prognosis for GBM patients treated with TMZ, we compared the prognostic efficacy between the TMZ response signature and *MGMT* promoter methylation. The TMZ response signature had greater prognostic value than *MGMT* promoter methylation in both datasets (*P* = 0.002 and 0.012, respectively) (Figures 4C,F).

### Nomogram Constructed for Predicting Individual Survival Rate

**Figure 5:**
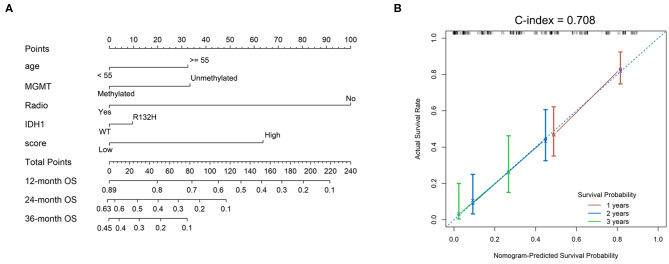
Nomograms to predict GBM patient survival. **(A)** Nomograms for predicting survival proportions of patients. **(B)** Plots depict the calibration of the TMZ response model in terms of agreement between predicted and observed 12-, 24-, and 36-month outcomes.

A nomogram using 186 selected GBMs was established according to the prognostic efficacies of clinicopathological parameters (Figure 5A). Factors, such as age, *MGMT* promoter status, radiation therapy, *IDH1* mutation status, and TMZ response-related score, were incorporated into the nomogram based on their two-level classification status to enable its clinical use. The nomogram illustrated that radiation therapy was the greatest contributor to prognosis, followed by the TMZ response-related score, *MGMT* promoter status, age, and *IDH* mutation status. We determined the concordance index to evaluate the effectiveness of our nomogram as 0.708. A calibration plot for the probability of survival at 1, 2, and 3 years was also drawn, which showed good agreement between the observed and predicted results (Figure 5B).

## Discussion

TMZ has been accepted as the standard and only chemotherapeutic agent for treating patients with GBM. However, biological heterogeneity among patients with GBM contributes to dramatically different treatment outcomes and is one of most critical barriers to individualized treatment and improved patient prognosis. Thus, determining whether a GBM patient is sensitive to TMZ is crucial for personalized therapy. Several studies have explored the TMZ resistance mechanism in GBM by inducing glioma cells to become insensitive to TMZ (19, 20). However, no studies have evaluated which GBM patients would benefit from TMZ therapy after neurosurgery. Thus, here, we systematically explored gene expression at the transcript level and biological processes significantly associated with glioma cell sensitivity to TMZ. In addition, we preliminarily demonstrated the synergistic effect of a combination treatment comprising TMZ and a telomerase inhibitor. Next, we constructed a gene expression signature comprising of six genes, *MROH8, BET1, PTPRN2, STC1, NKX3-1*, and *ARMC10*, which was strongly associated with the clinical characteristics of GBM and had good prognosis accuracy in both TCGA and the CGGA datasets.

Aberrant molecular changes, including *MGMT* overexpression induced by *MGMT* promoter unmethylation, have been shown to induce resistance to TMZ treatment by DNA damage repair (21, 22). In addition, previous studies found a close link between EMT, the Wnt signal pathway, and the TMZ resistance phenotype in gliomas (13, 23–25). These results were confirmed by Ho et al. (26) and our study. Moreover, in our two studies, we discovered that the immune response phenotype is strongly related to TMZ resistance at both the GBM tissue and cellular levels. In addition, GBMs that are primarily resistant to TMZ tend to have an immunosuppressive tumor microenvironment stimulated by NHE1 protein overexpression and inhibition of the NHE1-enhanced cytotoxic effects of TMZ, leading to enhanced tumor growth (27). Combined treatment with TMZ and a PD-1 antibody showed higher efficiency against gliomas both *in vitro* and in animal experiments compared to PD-1 antibody or TMZ therapy alone (28). However, the biological mechanisms underlying the synergistic effect of these two therapeutic agents in TMZ-resistant GBMs remain unclear. Additional studies, including clinical trials, should be performed to explore the mechanisms associated with these effects and the therapeutic regimens that should be followed.

A previous study suggested that *TERT* gene expression and telomerase activity are down-regulated in somewhat TMZ-sensitive glioma cells, whereas the inhibitory effect of TMZ against telomerase is weakened in TMZ-resistant glioma cells (29). These results indicate that telomerase is involved in TMZ resistance. Another study showed that inhibition of telomerase by an inactive, dominant-negative mutant of *hTERT* results in increased TMZ sensitivity in melanoma cells (30). In the present study, we validated the synergistic effect of TMZ combined with a telomerase inhibitor *in vitro* based on a bioinformatical analysis; our results were similar to those of previous studies. Clinical trials should be performed to evaluate the clinical benefit of combined treatment.

We also constructed a signature based on six genes identified as being related to TMZ resistance. *STC1* and *NKX3-1* have been demonstrated to enhance glioma cell proliferation, stemness, migration, and invasion (31–33). In addition, several studies have indicated that *PTPRN2* promotes the metastasis of breast cancer cells and that *ARMC10* plays a crucial role in mitochondrial dynamics (34, 35). These results suggest that inhibiting these genes would be efficient for reversing the TMZ resistance phenotype and suppressing glioma tumor growth; thus, they are ideal potential therapeutic targets for TMZ-resistant GBMs. In addition, the TMZ response signature identified in this study was strongly related to the clinical characteristics of GBMs. The signature also had a better prognostic efficacy than *MGMT* promoter methylation. More importantly, nomograms based on age, *MGMT* promoter status, radiation therapy, *IDH1* mutation status, and TMZ response-related score were found to be able to very accurately predict the individual survival probability of patients with GBM and, thus, provide guidance for adjuvant therapy following by neurosurgery.

## Conclusion

Overall, we systematically analyzed gene expression at the transcript level and biological processes significantly related to TMZ sensitivity. We discovered that the immune response phenotype is strongly related to TMZ resistance. We also found that telomerase is involved in TMZ resistance in GBM. Additionally, we identified several genes involved in the response to TMZ and constructed a signature comprising six genes, *MROH8, BET1, PTPRN2, STC1, NKX3-1*, and *ARMC10*, which are promising therapeutic targets for TMZ-resistant GBM. Finally, we established a nomogram based on GBM clinicopathological parameters that may provide guidance for adjuvant therapy followed by neurosurgery.

## Data Availability Statement

RNA-seq data used in this study were available from the Cancer Cell Line Encyclopedia (https://portals.broadinstitute.org/ccle/data). TMZ sensitivity data from human glioma cell lines were obtained from the Cancerrxgene database.

## Author Contributions

J-HW designed the study, analyzed the data, and revised the manuscript. H-QC, M-JZ, and A-SL designed the study, performed the experiments, analyzed the data and drafted the manuscript. H-JL, X-LM, and H-PQ collected data, designed the experiments, and analyzed the data.

## Conflict of Interest

The authors declare that the research was conducted in the absence of any commercial or financial relationships that could be construed as a potential conflict of interest.

## Supplementary Material

Supplementary Figure S1 The result of random survival forests variable hunting algorithm.

Supplementary Table S1 The detailed results of spearman analysis for 1373 TMZ response related genes.

Supplementary Table S2 The results of survival analysis for 307 protein-coding genes.
