# Supplementary figures and images for: Identifying Predictive Gene Expression and Signature Related to Temozolomide Sensitivity of Glioblastomas
Source: Front Oncol. 2020 May 22;10:669. doi: 10.3389/fonc.2020.00669 (PMC7258082; doi:10.3389/fonc.2020.00669)

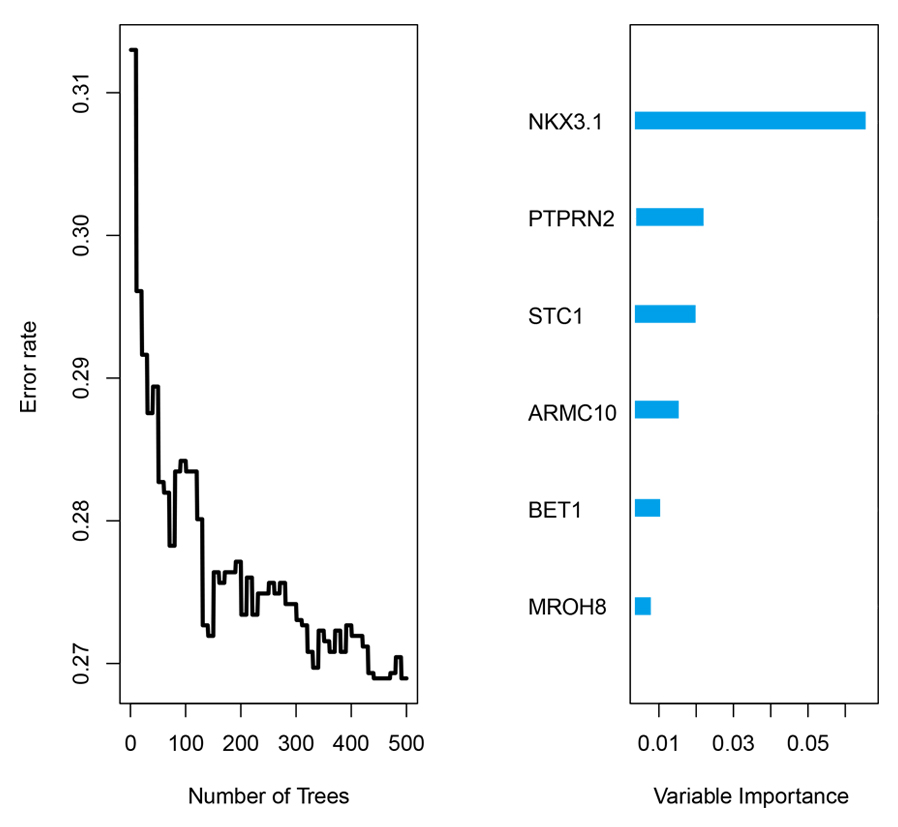

Supplement: Supplementary Figure S1 — The result of random survival forests variable hunting algorithm. [file Image_1.JPEG]
